# Supporting Carers: Study Protocol of a Meta-Review of Psychosocial Interventions for Carers of People With Cancer

**DOI:** 10.2196/56403

**Published:** 2024-09-13

**Authors:** Brona Nic Giolla Easpaig, Bronwyn Newman, Judith Johnson, Rebekah Laidsaar-Powell, Ursula M Sansom-Daly, Lucy Jones, Lukas Hofstätter, Eden G Robertson, Stephen Mears, Kabir Sattarshetty, Reema Harrison

**Affiliations:** 1 School of Nursing Charles Darwin University Sydney Australia; 2 Australian Institute for Health Innovation Macquarie University Sydney Australia; 3 School of Psychology University of Leeds Leeds United Kingdom; 4 Division of Nursing, Midwifery and Social Work School of Health Sciences University of Manchester Manchester United Kingdom; 5 Psycho-Oncology Co-Operative Group (PoCoG) School of Psychology, Faculty of Science, The University of Sydney Sydney Australia; 6 School of Clinical Medicine Discipline of Paediatrics & Child Health, UNSW Medicine and Health University of New South Wales Sydney Australia; 7 Kids Cancer Centre Sydney Children’s Hospital Sydney Australia; 8 Sydney Youth Cancer Service Nelune Comprehensive Cancer Centre, Prince of Wales Hospital Sydney Australia; 9 Neuroblastoma Australia Sydney Australia; 10 Carers New South Wales Sydney Australia; 11 Redkite Sydney Australia

**Keywords:** cancer, carer, caregiver, psychosocial, supportive intervention, psychosocial interventions, carers, caregivers, study protocol, well-being, wellbeing, end-users, evidence-based, evidence-based program, mental health

## Abstract

**Background:**

While there is a clear need for psychosocial interventions that promote the well-being of carers of patients with cancer, the corresponding evidence base is disparate, complex, and difficult for end users to navigate and interpret. Carers remain undersupported with a lack of dedicated, effective, evidence-based programs. We will conduct a meta-review to synthesize this evidence and determine the state of science in this field.

**Objective:**

This study aims to address the question, “what psychosocial interventions are available to promote the well-being of carers for people with cancer?”

**Methods:**

A meta-review will synthesize the relevant reviews of psychosocial interventions that have been developed and evaluated with carers for people with cancer. A total of 4 electronic databases (PsycInfo, MEDLINE, CINAHL, and Cochrane Database of Systematic Reviews) will be searched for reviews published between January 2013 and December 2023. A team-based approach will be taken for screening and assessment of the returned records against the eligibility criteria to determine inclusion. Included reviews will be critically appraised using the Joanna Briggs Institute Critical Appraisal Checklist for Systematic Reviews and Research Syntheses. Relevant data on study characteristics, carer and patient populations, intervention details, and psychosocial outcomes will be extracted, synthesized, and the findings will be presented in a narrative format.

**Results:**

It is anticipated that the study will be completed by October 2024.

**Conclusions:**

Ensuring that carers have access to evidence-based programs that promote their well-being as they care for loved ones is critical. This meta-review will contribute to program development and translation efforts by providing a clear picture of the intervention evidence base of carers of patients with cancer and identifying notable strengths, weaknesses, and gaps across the literature. The findings are anticipated to offer future directions to advance research in the field.

**Trial Registration:**

PROSPERO (CRD42023403219); https://tinyurl.com/4tnzv49s

**International Registered Report Identifier (IRRID):**

DERR1-10.2196/56403

## Introduction

### Background

Family and friend carers serve as the core, yet underacknowledged, members of the health team in coordinating and providing care for loved ones diagnosed with cancer [1]. The partners, parents, siblings, children, and friends of patients often fulfill this role, which spans pragmatic, clinical, and emotional domains of support [2-4]. Shifts in oncology care delivery, increasingly toward outpatient, community, and home settings have widened the scope of carers’ roles and responsibilities [1,4-6]. Carers of people with cancer may assume significant responsibilities in not only coordinating and organizing care, but also in providing direct clinical care (eg, administering medications) [4,6].

Becoming a carer is a role that many feel unprepared for, and are overwhelmed by, with implications for health and well-being [7,8]. Carers experience depression, anxiety, and distress, commonly at higher rates than the general population [9-11]. Furthermore, both the quality-of-care and clinical outcomes of the patient are linked to the well-being of carers [6]. There has been a growing interest in identifying psychosocial interventions that may be effective in supporting this population [7,12].

While there appears to be a large volume of literature reporting on studies of psychosocial interventions for carers of patients with cancer, this body of work is complex and fragmented, and it is challenging to draw clear conclusions about the evidence for specific types of programs or carer groups. The result is that carers remain undersupported, with limited evidence of the effective interventional approaches. Key issues within the evidence base of carers of patients with cancer that limit advancement in practice are the divergent scope and focus of current interventions (eg, in-person therapy for carers, web-based interventions for patient-spouse dyads [13], compared with psychological interventions for parents of children and adolescents with chronic illness [14]), all of which may be included in a single review.

A potential weakness in the current literature is that interventions have not necessarily been designed for carers as the target primary population; programs may be developed for patients and extended to include carers [15]. In such cases, carer-specific needs may not be met. In addition, the nature of relationships between carers and the person being cared for is not always sufficiently considered in the collation of intervention evidence. While findings suggest that distress and stress can arise while providing health care and with a loved one being ill [3,16], this appears underexplored. These limitations, with the divergences in the literature described, pose barriers for end users of this evidence [17].

### Review Aim and Question

Undertaking a meta-review was identified as a useful first step in addressing the abovementioned barriers to enable the development of targeted interventions that may offer greater impacts to reduce carer distress and enhance support [18]. A meta-review offers a means to develop an overall, coherent picture of a large volume of evidence [19,20], that is useful for those wishing to navigate this literature and identify the evidence relevant to them. This study aims to synthesize the evidence from reviews of psychosocial interventions designed to support the well-being of carers of people with cancer.

This study will address the question, “what psychosocial interventions are available to promote the well-being of carers for people with cancer, as reported in the evidence from reviews?”

## Methods

### Overview

Please revert back to the original text or alternatively revise to read: “A meta-review was selected as a method that offers a systematic and rigorous approach to the identification and review of relevant evidence in the form of various types of reviews [21]. The protocol for this study is registered with the PROSPERO (International Prospective Register of Systematic Reviews; CRD42023403219). In the absence of a method-specific protocol reporting framework, the PRISMA-P (Preferred Reporting Items for Systematic Reviews and Meta-Analysis Protocols) guidelines are used to report this study protocol (Multimedia Appendix 1) [22].

### Eligibility Criteria

The following eligibility criteria will be used to determine study inclusion (Textbox 1).

**Eligibility criteria.**

**Inclusion criteria**

- Population
  - Carer for a family member (adult or child) or a person with whom they have a personal relationship who has received a diagnosis of cancer.
  - People who are providing care on an “informal” basis, which they have not been professionally contracted to provide.
- Intervention
  - Nonpharmacological interventions that are designed to positively impact some aspect of the psychosocial well-being of carers of people with cancer (such as interventions focused on improving mental health, health-related quality of life, or life satisfaction).
  - Psychosocial interventions can include educational, informational, and therapeutic activities designed to promote well-being (eg, cognitive behavioral therapy).
  - Remote, in-person, and hybrid modes of intervention delivery will be eligible for inclusion.
  - Both facilitated and self-directed interventions will be eligible for inclusion.
- Outcome
  - Primary
    - Psychosocial outcomes of carers: quality of life, depression, anxiety, resilience, psychological distress, and any other psychosocial outcomes reported on.
  - Secondary
    - Patient or family well-being outcomes associated with the intervention (eg, patient psychological distress).
    - Implementation-related outcomes, such as acceptability, feasibility, and uptake into practice.
- Context
  - Care in the home, community, health care, or any other setting.
- Study methods
  - Any type of review (eg, scoping reviews, narrative reviews, integrated reviews, systematic reviews, meta-reviews, reviews of qualitative evidence, meta-syntheses, and meta-analyses) of interventions including controlled trials, quasi-experimental studies, pilot studies, feasibility studies, pre-post studies, and evaluation studies.
  - Reviews of studies using any methods are eligible—quantitative, qualitative, and mixed- and multimethods.
- Publication type
  - Peer-reviewed publications.
- Publication date
  - January 1, 2013-December 31, 2023.
- Language
  - English.

**Exclusion criteria**

- Intervention
  - Interventions in which psychosocial well-being (eg, reduced psychological distress and improved quality of life) is not a stated outcome.
  - Studies that do not report the data and results separately for carers of people with cancer.
- Study methods
  - Nonreview studies.
- Publication type
  - Conference abstracts, editorials, opinion pieces, non–peer-reviewed research, and nonempirical research will be excluded.

### Information Sources

Systematic searching of PsycInfo, MEDLINE, CINAHL, and Cochrane Database of Systematic Reviews will be performed. In addition, the reference lists of relevant reviews will be audited to identify other potentially eligible reviews. The search will cover a 10-year period from January 1, 2013, to December 31, 2023.

### Search Strategy

The search strategy for the databases listed was developed through consultation with a medical research librarian. The search was developed based on the search strategy used in the study by Treanor et al [23] Cochrane review of the psychosocial interventions for informal carers of people living with cancer and informed by concepts encompassed in Fletcher et al [24] model of the cancer family carer experience. Search terms were updated as required, including terms related to the study type (eg, “systematic review”). The search period was selected to identify recently published reviews and capture the current evidence landscape. The finalized search strategy uses a combination of MeSH (Medical Subject Headings) terms and keywords and as an example, the strategy developed for the MEDLINE database is included in Multimedia Appendix 2.

### Selection Process

Records retrieved from the searches will be imported into EndNote X9 (Clarivate, citation management software) [25], and duplicates will be subsequently removed. The remaining records will then be uploaded to Covidence (literature review management tool) [26], which will be used to manage the screening of records. The titles, abstracts, and keywords of records will be screened by 1 of the team members against the criteria to determine inclusion. The full texts for included records will then be retrieved and 2 team members will independently assess each text against the eligibility criteria. Disagreements will be resolved by team discussion, with discrepancies discussed with a third team member until a resolution is reached. The search results will be documented and reported using the PRISMA (Preferred Reporting Items for Systematic Reviews and Meta-Analyses) guidelines [27], with adaptations made as needed to reflect the meta-review method.

### Data Collection Process

A draft data extraction template will be developed, and 2 team members will independently extract data for a shared 10% of the included reviews to identify any amendments needed to the template. Once finalized, a team-based approach to data extraction will be taken where data will be extracted by one team member, all of which will be subsequently cross-checked by another member of the team. Discrepancies will be resolved through discussion. Microsoft Excel will be used to manage the data extraction process [28]. Data will be extracted in the areas of study characteristics, populations of carers and patients, intervention details, and reported impact (if any) on the outcomes of interest (Table 1).

**Table 1:** Data items for extraction.

Areas of data collection		Data item details
Study characteristics		Year, review aims, types of review, any geographical restrictions of review, conditions of those cared for methods, study types and designs included in review, total number of included studies (and articles, if different), participants’ total number or sample size details, search period, synthesis method, critical and quality appraisal tool used, and any other notable details
Population		Population focused in the review, target carer population, target, patient population, and any other notable details
Intervention details		Details about intervention development, mode of delivery, theoretical bases, settings, facilitators, details about frequency, duration or length of interventions, and any other notable details
**Outcomes of interest**		
	Carer psychosocial outcomes	Quality of life, depression, anxiety, resilience, psychological distress, and any other psychosocial outcomes reported on
	Implementation-related outcomes	Acceptability, feasibility and uptake into practice, and other implementation-relevant outcomes
	Patient or family well-being outcomes	Patient or family well-being outcomes associated with the intervention such as patient psychological distress

### Data Synthesis

The extracted data will be collated and organized. A narrative approach will be taken to describe the results, study characteristics, populations, interventions, outcomes, and any other details of interest. Data will be categorized and grouped (eg, by types of intervention facilitators) and where possible, a quantitative description will be provided (eg, the total number of studies reported across reviews). The narrative will provide a mapping and organization of the data to promote a coherent picture of the body of evidence. Data permitting salient groupings and dimensions of differences in the evidence, such as types of interventions, populations, and outcomes will be explored. For example, a reader would be able to glean the evidence for psychosocial interventions for couples.

### Critical Appraisal

The risk of bias and quality of methodological results for the included reviews will be evaluated using a standardized appraisal tool specifically designed for the appraisal of systematic reviews and research syntheses [29]. One team member will initially conduct an appraisal that will be cross-checked by a second team member. Any discrepancies will be discussed by team members and resolved.

### Ethical Considerations

Ethical approval is not required for this manuscript. The manuscript does not contain any data.

## Results

To date, the search and study selection process is underway, with a search to be rerun in January 2024 to encompass the full search period. A preliminary extraction method has been developed, tested, and discussed among the team to help refine the process. It is anticipated that the study will be completed by October 2024.

## Discussion

### Principal Findings

The study outlined in this paper is anticipated to reveal the state of the science of cancer caregiver psychosocial support interventions. While informative meta-review research has been conducted in related areas (eg, a more broadly focused study of support for carers of people with a wide range of conditions [30], or a review specifically focused on family-based interventions in palliative cancer care [31]), these studies were not targeted to address the aim of the current research. The proposed synthesis of the evidence from reviews of psychosocial interventions designed to support the well-being of carers of people with cancer is expected to result in the formation of a coherent picture of a vast and fragmented literature base. These findings will offer a novel and valuable point of view conducive to understanding the quality of this evidence, the strengths, and weaknesses, and identify research priorities for supporting evidence-based programs.

Attention in the synthesis to the grouping of particular outcomes, carer populations, and intervention characteristics (and any other important groupings) will help end users identify the review evidence most pertinent to them, in addition to serving as a useful foundation for others interested in further studying the evidence about particular carer populations or interventions. The findings from this meta-review will be reported in a scholarly publication, presented at conferences, and disseminated through professional networks. Any variations to the protocol will be advised of in the record of the protocol and reports of the findings.

### Strengths and Limitations

This is a well-designed study that had input in the development of the search strategy from a research librarian in addition to team members’ subject matter and methodological and clinical expertise. The process is guided by an established method with inbuilt procedures to promote rigor and transparency. Meta-review findings are limited by the quality of the included reviews and the primary research reported therein [17,18,32]. Furthermore, as the study is designed to capture review evidence, this means that recent findings reported in newly published primary research will not be captured [33].

### Conclusion

There is a clear need to ensure that carers have access to evidence-based programs that can effectively support their well-being as they care for their loved ones. Current models of cancer care rely heavily on the work of carers, and, given the growing burden of cancer worldwide [30], this caregiving work is also vital to health system sustainability. This meta-review will facilitate an improved understanding of the evidence base, enabling better identification of research strengths, limitations, and gaps. It will also enhance the navigation of the literature, allowing researchers, clinicians, and policymakers to more readily review evidence relevant to them [18,19], in turn supporting the translation of evidence into practice.

## Appendix

Multimedia Appendix 1 PRISMA-P (Preferred Reporting Items for Systematic Reviews and Meta-Analyses Protocols) checklist.

Multimedia Appendix 2 Search Strategy.

## Abbreviations

MeSH: Medical Subject Headings
PRISMA: Preferred Reporting Items for Systematic Reviews and Meta-Analyses Reviews
PRISMA- P: Preferred Reporting Items for Systematic Reviews and Meta-Analyses Protocols
PROSPERO: International Prospective Register of Systematic Reviews

## Data Availability

All data used during this study will be included in this published article and its Multimedia Appendices.
